# Supplementary material for: Dislocation Majorana bound states in iron-based superconductors
Source: Nat Commun. 2024 Mar 15;15:2337. doi: 10.1038/s41467-024-46618-9 (PMC10943028; doi:10.1038/s41467-024-46618-9)
Supplement: Supplementary file 1 — Supplementary Information [file 41467_2024_46618_MOESM1_ESM.pdf]

Supplementary Information for "Dislocation Majorana Bound States in Iron-based Superconductors"

Lun-Hui Hu[1,2,4] and Rui-Xing Zhang[1,3,2,*]
[1]*Department of Physics and Astronomy, The University of Tennessee, Knoxville, Tennessee 37996, USA*
[2]*Institute for Advanced Materials and Manufacturing, The University of Tennessee, Knoxville, Tennessee 37920, USA*
[3]*Department of Materials Science and Engineering, The University of Tennessee, Knoxville, Tennessee 37996, USA*
[4]*Center for Correlated Matter and School of Physics, Zhejiang University, Hangzhou 310058, China and*
[*]correspondence to ruixing@utk.edu

Contents

1. Localization Length of Dislocation Majorana Bound States 1
   A. Analytical theory for nested domain wall construction 1
      1. Solution for Dirac surface states 2
      2. Solution for 1D chiral Majorana hinge modes (MHMs) 3
      3. Wavefunction for dMBS 4
   B. Numerical approach I: finite-size gap 5
   C. Numerical approach II: wave functions 6

2. Surface Topological Phase Diagram for $FeTe_{1-x}Se_x$: a First-Principles-Based Model Study 8
   A. Eight-band model Hamiltonian and parameters 9
   B. Superconducting band structures 10
   C. Surface topological phase diagrams 11
   D. In-plane magnetism induced partial Fermi surfaces 12

3. Robustness of dMBS Against Magnetic Disorders 13

Supplementary Note. 1 Localization Length of Dislocation Majorana Bound States

In this section, we employ a combination of analytical and numerical approaches to investigate the localization length of dislocation Majorana bound states (dMBSs). We establish its relationship with three parameters, namely the strength of spin-orbit coupling (v), surface magnetism (M_z), and the superconducting gap (Δ_0).

A. Analytical theory for nested domain wall construction

The logic is briefly illustrated as follows. We first solve the surface Dirac states for the four-band topological insulator model, then project the surface magnetism and bulk superconductivity onto the surface Dirac states. By building the effective model for our nested domain wall picture [see Fig. 1 in the main text], we can extract the localization length information.

The minimal Bogoliubov-de Gennes (BdG) Hamiltonian used in the main text for $FeTe_{1-x}Se_x$ is

$$\mathcal{H}_{\mathrm{BdG}}(\mathbf{k}) = \begin{pmatrix} \mathcal{H}_0(\mathbf{k}) - \mu & \Delta \\ \Delta^\dagger & -\mathcal{H}_0^*(-\mathbf{k}) + \mu \end{pmatrix}, \tag{1}$$

where the normal-state Hamiltonian

$$\mathcal{H}_0 = v(\sin k_y \Gamma_1 - \sin k_x \Gamma_2 + \sin k_z \Gamma_4) + m(\mathbf{k})\Gamma_5, \tag{2}$$

where $m(\mathbf{k}) = m_0 - m_1(\cos k_x + \cos k_y) - m_2 \cos k_z$ and μ is the chemical potential. The Γ matrices are defined as

$$\Gamma_1 = \sigma_x \otimes s_x, \Gamma_2 = \sigma_x \otimes s_y, \Gamma_3 = \sigma_x \otimes s_z, \Gamma_4 = \sigma_y \otimes s_0, \Gamma_5 = \sigma_z \otimes s_0, \tag{3}$$

where $s_{0,x,y,z}$ and $\sigma_{0,x,y,z}$ are Pauli matrices for spin and orbital d.o.f., respectively. We choose $v = 1, m_0 = -4, m_1 = -2, m_2 = 1$ to ensure a single topological band inversion at Z, leading to $\nu_0 = 1$ and $\boldsymbol{\nu} = (0, 0, 1)$. Hereafter, we focus on the s-wave intra-orbital spin-singlet pairing potential $\Delta = \Delta_0(i\sigma_0 \otimes s_y)$.

1. *Solution for Dirac surface states*

Following Ref. [1], we solve the normal-state Hamiltonian in the continue limit by plunging

$$\sin k_i \to k_i \text{ for } i = x, y, z, \tag{4a}$$

$$\cos k_i \to 1 - \frac{1}{2}k_i^2 \text{ for } i = x, y, \tag{4b}$$

$$\cos k_z \to -1 + \frac{1}{2}k_z^2. \tag{4c}$$

into Eq. (2). We also separate it into two parts

$$\mathcal{H}_0 = \mathcal{H}_{0,xy} + \mathcal{H}_{0z}, \tag{5}$$

where $\mathcal{H}_{0,xy} = \frac{m_1}{2}(k_x^2 + k_y^2)\Gamma_5 + v(k_y\Gamma_1 - k_x\Gamma_2)$ and $\mathcal{H}_{0,z} = (m_0 z - \frac{1}{2}m_2 k_z^2)\Gamma_5 + v k_z \Gamma_4$ with $m_{0z} = m_0 - 2m_1 + m_2$. In the main text, we have employed the parameter values $m_{0z} = m_2 = 1$, resulting in $\mathcal{H}_{0z}$ taking the form of a 1D massive Dirac equation, as depicted in Fig. 1 (a). The chiral symmetry inherent in $\mathcal{H}_{0z}$ guarantees the existence of a zero-energy localized solution. Using this solution as a basis, we project $\mathcal{H}_{0,xy}$ onto the Dirac surface Hamiltonian. Additionally, we observe that

$$\mathcal{H}_{0z} = \mathcal{H}_{0z}^{\uparrow} \oplus \mathcal{H}_{0z}^{\downarrow}, \tag{6}$$

where $\mathcal{H}_{0z}^{\uparrow} = \mathcal{H}_{0z}^{\downarrow} = (m_0 z - \frac{1}{2}m_2 k_z^2)\sigma_z + v k_z \sigma_y$. They have the same chiral symmetry $\{\sigma_x, \mathcal{H}_{0z}^{\uparrow}\} = \{\sigma_x, \mathcal{H}_{0z}^{\downarrow}\} = 0$. We only need to solve $\mathcal{H}_{0z}^{\uparrow}$, whose zero-energy solution should be eigen-state of σ_x. The trial wave function can be

$$\psi(z) = a e^{\lambda z}|\phi_+\rangle + c e^{\lambda' z}|\phi_-\rangle, \tag{7}$$

where $\sigma_x|\phi_\pm\rangle = \pm|\phi_\pm\rangle$. From $\mathcal{H}_{0z}^{\uparrow}(k_z \to -i\partial_z)\psi(z) = 0$, we get the solution for λ and λ',

$$\lambda_{1,2} = -\lambda'_{1,2} = \frac{1}{m_2}[v \pm \sqrt{v^2 - 2m_{0z}m_2}] \tag{8}$$

which gives rise to a generic zero-energy wave function

$$\psi(z) = (a e^{\lambda_1 z} + b e^{\lambda_2 z})|\phi_+\rangle + (c e^{-\lambda_1 z} + d e^{-\lambda_2 z})|\phi_-\rangle. \tag{9}$$

Recall the parameters we used, $v > 0$, so that $\mathrm{Re}[\lambda_{1,2}] > 0$. Therefore, the localized mode near $z = 0$ boundary between sample ($z >= 0$) and vacuum ($z < 0$) should be

$$\psi(z) = c(e^{-\lambda_1 z} - e^{-\lambda_2 z})|\phi_-\rangle, \tag{10}$$

where $c + d = 0$ has been used because of the boundary condition $\psi(z = 0) = 0$, and $|c| = \sqrt{\frac{A(A^2+B^2)}{2A^2+B^2}}$ is the normalization factor with $A = \frac{v}{2m_2}$ and $B = \sqrt{2m_{0z}m_2 - v^2}$. Notice that $\lambda_{1,2} = A \pm iB$. Therefore, we obtain the Dirac surface states' wave function for $\mathcal{H}_{0z}$,

$$\psi^{\uparrow}(z) = 2c e^{-Az}\sin(Bz)|\phi_-\rangle \otimes |\uparrow\rangle, \tag{11a}$$

$$\psi^{\downarrow}(z) = 2c e^{-Az}\sin(Bz)|\phi_-\rangle \otimes |\downarrow\rangle. \tag{11b}$$

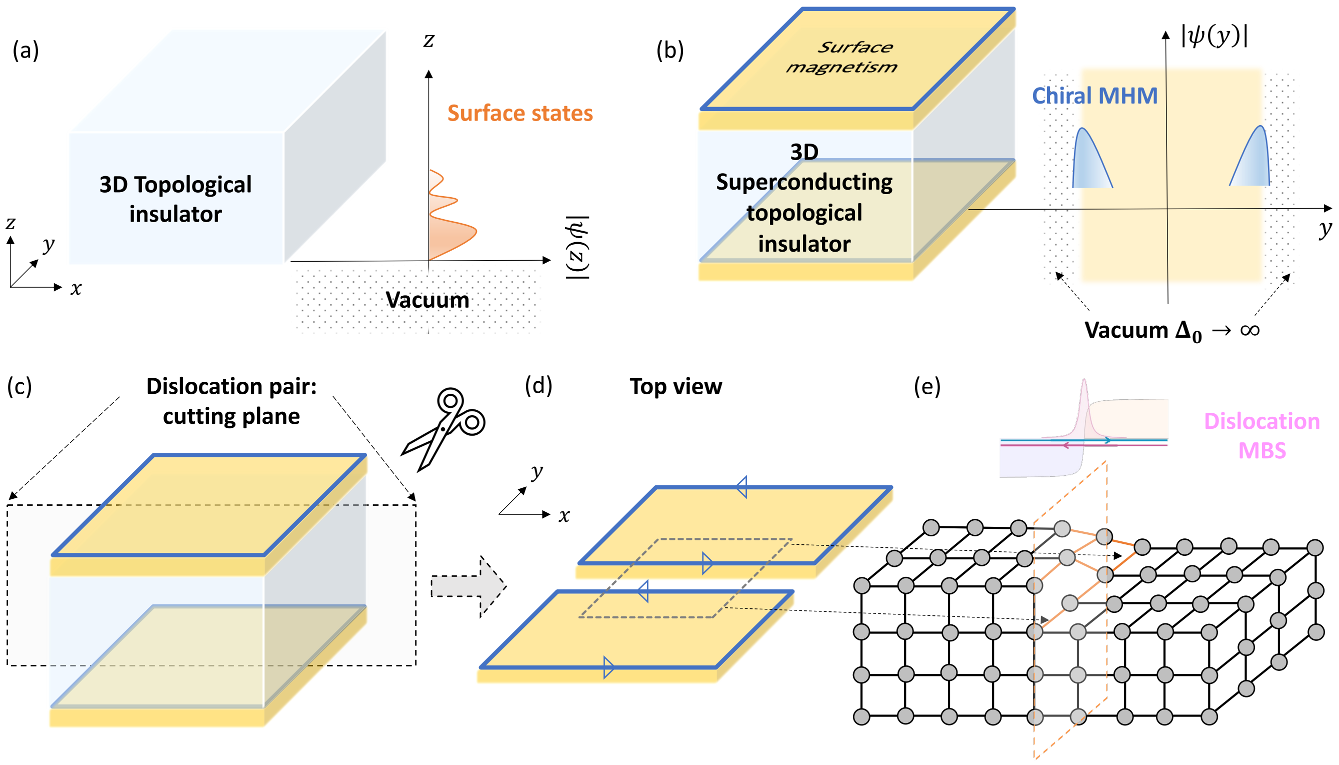

Supplementary Figure 1. Schematic for analytically solving surface states, chiral Majorana hinge modes, and dislocation Majorana modes. (a) illustrates the localized Dirac surface states of a topological insulator. (b) showcases the interplay between surface magnetism and bulk superconductivity, resulting in chiral MHMs represented by blue lines along the hinges of the top and bottom surfaces. (c) displays the cutting plane formed by a pair of dislocation lines. The corresponding top view is also presented on the top surface in (d), where each side features one chiral MHM mode (the triangle indicates the propagating direction). The final step involves gluing these two sections together to restore the lattice, leading to the hybridization between the two chiral MHMs and the emergence of 0D dMBSs, as schematically shown in (e).

The spinor part provides the basis for Dirac surface states, $\{|\phi_-\rangle \otimes |\uparrow\rangle, |\phi_-\rangle \otimes |\downarrow\rangle\}$, where $\sigma_x|\phi_\pm\rangle = \pm|\phi_\pm\rangle$ leads to $|\phi_+\rangle = \frac{1}{\sqrt{2}}(1,1)^T$ and $|\phi_-\rangle = \frac{1}{\sqrt{2}}(1,-1)^T$. We next use the first-order perturbation for the surface state Hamiltonian up to linear k order,

$$\mathcal{H}_{surf} = v(k_x s_y - k_y s_x). \tag{12}$$

Please note that $\mathbf{s}$ represents the Pauli matrices for the spin degree of freedom. The localization length for the surface state is determined by $\xi_{\text{surf}} = 1/A = 2m_2/v$, signifying that a small spin-orbit coupling (v) will leads to a large localization length. This observation further can explain the large localization length of Dirac surface states in iron-based superconductors, which will be discussed later (see Fig. 6 (c) below).

2. *Solution for 1D chiral Majorana hinge modes (MHMs)*

Next, we consider the competition between surface magnetism (spin polarization along z direction) and s-wave bulk superconductivity. The BdG Hamiltonian for top surface of the sample can be described as

$$\mathcal{H}_{BdG} = v(k_y s_y \gamma_z - k_x s_x \gamma_0) - \mu s_0 \gamma_z + M_z s_z \gamma_z + \Delta_0 s_y \gamma_y, \tag{13}$$

where γ are Pauli matrices for the particle-hole degree of freedom. The particle-hole symmetry is $P = s_0\gamma_x K$ with K the complex conjugate and the two-fold $C_{2z} = is_z\gamma_z$. Without loss of generality, we consider both $M_z > 0$ and $\Delta_0 > 0$. The gap closing happens at $\mu^2 + \Delta_0^2 = M_z^2$, and a topological gap is given by $\Delta_{topo} = M_z - \sqrt{\mu^2 + \Delta_0}$. For simplicity, we work on $\mu = 0$, because we are going to discuss the relationship between the localization length of dMBSs and tuning parameters v, M_z, and Δ_0. A nonzero μ does not quantitatively change the main results.

We then use the same trick for the solution of zero-energy of the topological insulator model, and first set $k_x \to 0$ and $k_y \to -i\partial_y$ for the surface BdG Hamiltonian $\mathcal{H}_{BdG}$. The chiral symmetry is defined as $\{s_y\gamma_z, \mathcal{H}_{BdG}(k_x = 0, -i\partial_y)\} = 0$. To obtain the chiral MHM wave function, we consider the domain wall constrain, as illustrated in Fig. 1 (b). On the surface of the sample, we consider a topological region, $M_z > \Delta_0$. In the vacuum region, we can effectively set $\Delta_0 \to \infty$ so it must be trivial. For a trial wave function $\psi(y) = e^{\lambda y}|\phi\rangle$, we have the scalar equation for λ,

$$\det[(iv\lambda)s_x\gamma_0 + M_z s_z\gamma_z + \Delta_0 s_y\gamma_y] = 0, \tag{14}$$

which gives rise to $(M_z - \Delta_0 - v\lambda)(M_z + \Delta_0 - v\lambda)(M_z - \Delta_0 + v\lambda)(M_z + \Delta_0 + v\lambda) = 0$. Considering the domain wall constrain, the solution $\lambda = \frac{1}{v}(-M_z + \Delta_0)$ is for the localized zero-energy state. It can be checked $\lambda < 0$ for the $y > 0$ region (topological with $M_z > \Delta_0$), while $\lambda \to +\infty$ for the $y < 0$ region (trivial vacuum). Therefore, the chiral MHM solution for $\mathcal{H}_{BdG}(k_x = 0, -i\partial_y)$ is

$$\psi(y) = ae^{(-M_z+\Delta_0)y/v}|\phi_+\rangle, \tag{15}$$

where a is the normalization factor and the spinor part is

$$|\phi_+\rangle = \frac{1}{2}(1, -i, -i, 1)^T. \tag{16}$$

It is simultaneously an eigen-state of both chiral symmetry $s_y\gamma_z|\phi_+\rangle = -|\phi_+\rangle$ and the particle-hole symmetry $s_0\gamma_x K|\phi_+\rangle = i|\phi_+\rangle$. It is also an eigen-state of the k_x term (i.e., $k_x s_x\gamma_0$ in $\mathcal{H}_{Bdg}$), which gives rise to the dispersion of 1D chiral MHM along the 1D boundary of the sample surface,

$$\langle\phi_+|k_x s_y\gamma_z|\phi_+\rangle = k_x. \tag{17}$$

3. *Wavefunction for dMBS*

We next solve the dMBS by inserting a pair of dislocation lines to the system. As we discussed in the main text, the dislocation line is aligned to the z-direction. There are two main steps.

1. Cut the sample into two parts by the cutting plane expanded by this pair of dislocation lines, as illustrated in Fig. 1 (c) [see the dashed rectangle]. The 1D chiral MHM is also divided into two parts, and near the touching edges, those two 1D chiral MHMs propagate along different direction due to the C_{2z} symmetry [see Fig. 1 (d)].

2. Glue these two 1D chiral MHMs by restoring the lattice, as illustrated in Fig. 1 (e).

After these two steps, we can build an effective two-by-two Hamiltonian consisting of two oppositely propagating 1D chiral MHMs, which are

$$\psi_R(y) \approx e^{(-M_z+\Delta_0)y/v}(1, -i, -i, 1)^T, \tag{18a}$$

$$\psi_L(y) \approx e^{(M_z-\Delta_0)y/v}(1, i, i, 1)^T. \tag{18b}$$

This gives rise to

$$\mathcal{H}_{dis} = vk_x\tau_z + \mathrm{Im}[t_c]\tau_y + \mathrm{Re}[t_c]\tau_x, \tag{19}$$

where the inter-edge coupling t_c is due to the direct hopping ($vk_y s_x\gamma_0 \to -iv\partial_y s_x\gamma_0$),

$$t_c \approx \int_{-\Delta_y}^{\Delta_y} dy\langle\phi_R|s_x\gamma_0|\phi_L\rangle \times \left(e^{(-M_z+\Delta_0)y/v}[-iv\partial_y]e^{(M_z-\Delta_0)y/v}\right) \propto -iv\frac{M_z - \Delta_0}{v} = -i(M_z - \Delta_0). \tag{20}$$

Please also note that t_c is just a constant if there is no dislocation pairs, however, its sign depends on the position [2] for the dislocation case. For example, these two dislocation lines are separated in real space and locate at $(N_{cx}, N_{cy}, z = 1) \to (N_{cx}, N_{cy}, z = N_z)$ [line 1] and $(N_{cx} + \Delta_x, N_{cy}, z = 1) \to (N_{cx} + \Delta_x, N_{cy}, z = N_z)$ [line 2]. The in-plane distance between these two dislocation lines are given by Δ_x. Then, we have

$$t_c = \begin{cases} x \le N_{cx} \text{ or } x \ge N_{cx} + \Delta_x, & \text{direct in-plane hopping: } -i(M_z - \Delta_0), \\ N_{cx} \ge x \le N_{cx} + \Delta_x, & \text{inter-layer hopping: } -i(M_z - \Delta_0) \times (-1). \end{cases} \tag{21}$$

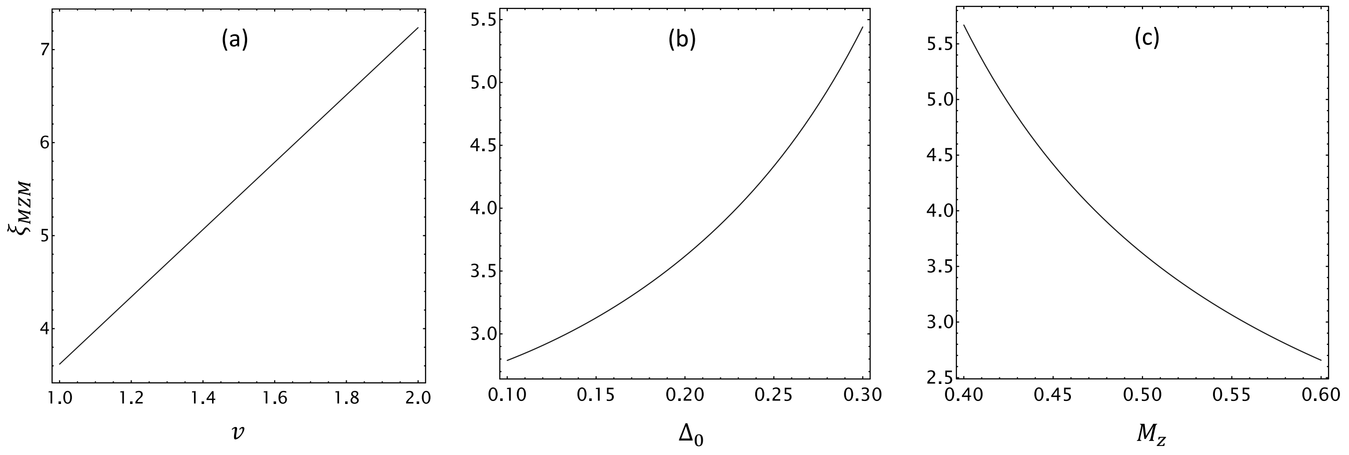

Supplementary Figure 2. **The analytical result for the localization length (ξ_{MZM}) of dMBSs.** We illustrate the relationship between ξ_{MZM} and three key parameters: the Dirac surface state's velocity ν in (a), the proximity-induced superconducting gap Δ_0 in (b), and the surface magnetization strength M_z in (c).

The hopping associated with spin-orbit coupling undergoes a π phase shift when circling the dislocation, explaining the negative sign above. Consequently, t_c serves as the mass term for the two 1D chiral MHMs, further leading to a 1D mass domain wall along the line connecting these two dislocation lines. This necessarily leads to a 0D Majorana zero mode (MZM), referred to as a dMBS in the main text. Similar to our discussion in Sec. 1 A 1, the localization length of dMBS is given by

$$\xi_{MZM} \approx \frac{v}{M_z - \Delta_0}. \tag{22}$$

This implies that ξ_{MZM} increases with an increase in either v or Δ_0, keeping in mind the requirement $M_z > \Delta_0$. Additionally, ξ_{MZM} increases with a decrease in M_z. The introduction of a finite chemical potential μ only modifies the topological gap. Consequently, we can argue that

$$\xi_{MZM} \approx \frac{v}{M_z - \sqrt{\Delta_0^2 + \mu^2}}. \tag{23}$$

Obviously, it does not change the main conclusion, note that $M_z > \sqrt{\Delta_0^2 + \mu^2}$ is required. A plot of Eq. (23) is show in Fig. 2 for illustrations. Please note that Eq. (23) is obtained from a nested domain wall construction, and thus it can only qualitatively capture the in-plane localization.

We will confirm this analytical result by comprehensive numerical simulations. In the following, we will discuss two methods [see Fig. 3] to do that and both methods show qualitatively the same results, which will be discussed in detail as follows.

B. Numerical approach I: finite-size gap

In this section, we use numerical simulation for extracting the finite-size gap Δ_{MZM} of a pair of dMBSS based on the exact diagonalization of the 3D lattice Hamiltonian. As shown in Fig. 3 (a), we only consider top surface magnetism and realize two dMBSs localized at the end of each dislocation line. The two gray cones in Fig. 3 (a) present the dMBSs. This simplification can help to reduce the lattice size along the z direction, thus, thereby minimizing the memory usage in numerical simulations. Therefore, the finite size gap Δ_{MZM} is mainly caused by the hybridization between the two top dMBSs, providing relatively accurate in-plane localization length of MZMs. By varying the in-plane distance between these two dislocation lines, labeled by Δ_x in Fig. 3 (a), we can obtain the curve for Δ_{MZM} as a function of Δ_x, as shown in Fig. 3 (b) for an example.

In the numerical calculation, we set $N_y = 2N_x = 2\Delta_x \in [28, 56]$ and $N_z = 16$. Even though the calculation on the lattice size with $N_y = 2N_x = 56$ and $N_z = 16$ almost reaches our numerical computing ability, this method shows enough data to fit ξ_{MZM}. An example is shown in Fig. 3 (b), where the blue dots represent numerical results, and can be fitted by an exponential function,

$$\Delta_{MZM} \approx a e^{-\Delta_x/\xi_{MZM}}. \tag{24}$$

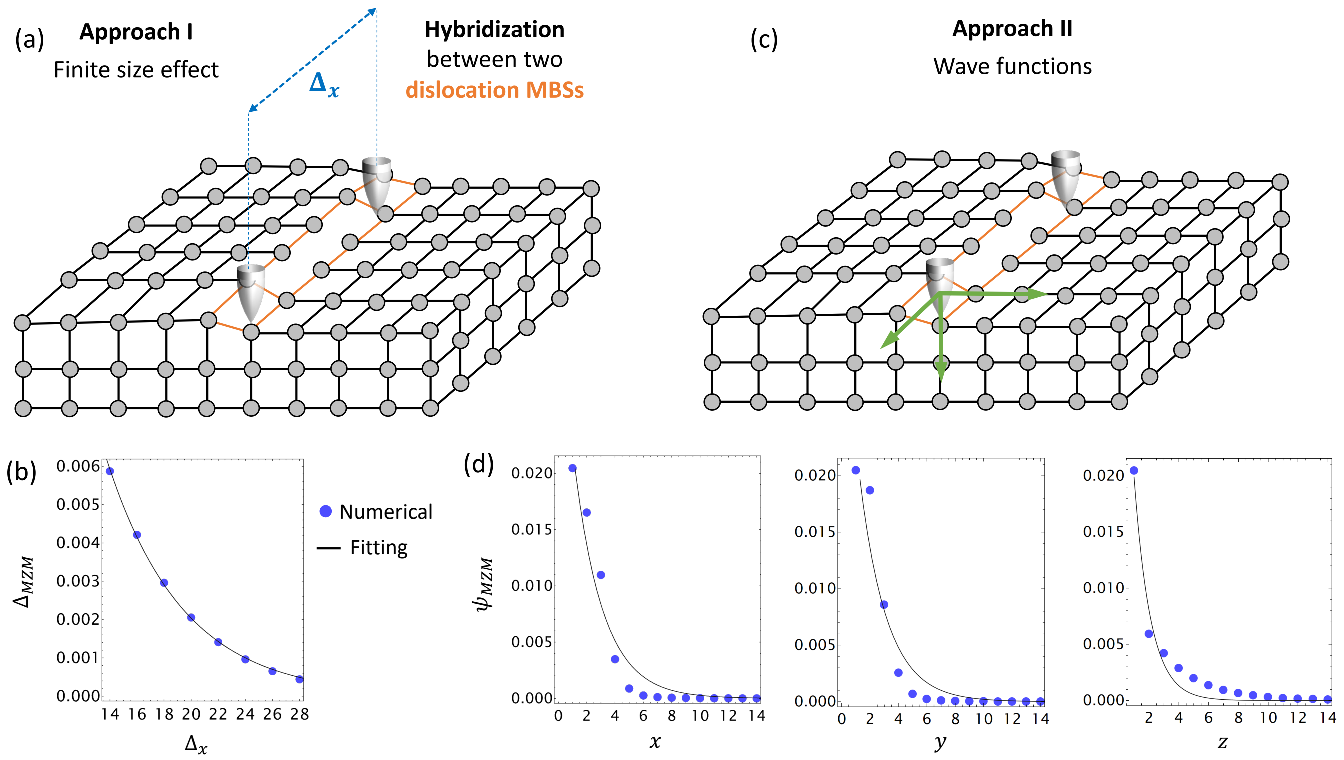

Supplementary Figure 3. **Numerically extracting the localization length (ξ_{MZM}) of dMBSs.** (a) represents the approach I (finite-size effect) by numerical calculating the hybridization gap Δ_{MZM} between two dMBSs. For example, the corresponding calculation is presented in (b), showing Δ_{MZM} as a function of the in-plane distance between them, denoted as Δ_x. The numerical results are represented by blue dots, and the black line indicates the fitting result. From this fit, we extract $\xi_{MZM} \sim 5.62057$ in the unit of the in-plane lattice constant. Furthermore, (c) demonstrates approach II by extracting ξ_{MZM} along various directions from the wave function distribution of a single dMBS, as depicted in (d). Likewise, the numerical results are represented by blue dots, and the black line indicates the fitting result.

For the result in Fig. 3 (b), the black line is for the fitting results and shows the in-plane localization length of MZMs as $\xi_{MZM} = 5.62057$ in unit of in-plane lattice constant. It shows our fitting demonstrates satisfactory accuracy.

This is our numerical approach I by calculating the finite-size gap. Based on this, we can further calculate the dependence of ξ_{MZM} on tuning parameters, including spin-orbit coupling v, superconducting gap Δ_0, and surface magnetism M_z. Please notice that M_z and Δ_0 here are directly added in the 3D bulk lattice Hamiltonian, which can be slightly different from the corresponding projected values for Dirac surface states, as used in the analytical approach for localization length. The numerical results are shown in Fig. 4, which shows a great agreement with analytical results in Eq. (23) [also see Fig. 2]. Increasing both v and Δ_0 gives rise to the increase of ξ_{MZM}, but increasing M_z leads to the decrease of ξ_{MZM}.

C. Numerical approach II: wave functions

In this section, we use numerical simulation for extracting the amplitude of the Majorana wave function distribution ψ_{MZM} based on the exact diagonalization of the 3D lattice Hamiltonian. This approach, however, is not as efficient as the previous finite-size-effect approach in terms of extracting ξ_{MZM}. Yet, we still decide to include our numerical findings for completeness and comparison. To be specific, we consider two slightly different scenarios:

1. Similar to the approach I, we only consider top surface magnetism.

2. We consider magnetism for both top and bottom surfaces.

In both situations, we take ψ_{MZM} for one of those MZMs [gray cones in Fig. 3 (c)], and numerically extract ψ_{MZM} along x, y and z directions, which enables the fitting of the localization length ξ_{MZM} along these three directions. Note that the dislocation line end is set to be origin for ψ_{MZM}. In the numerical simulation, we fix the lattice size

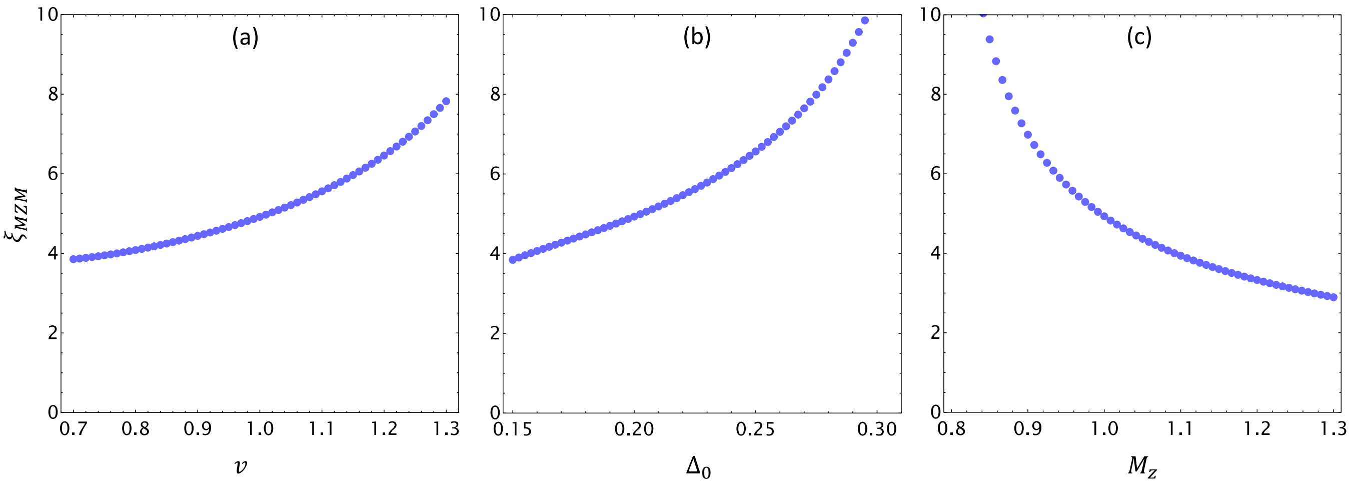

Supplementary Figure 4. **The numerical results for the localization length (ξ_{MZM}) of dMBSs based on wave-function overlaps.** Similar to the analytical results of ξ_{MZM} shown in Fig. 2 above, here we use the approach I to numerically calculate ξ_{MZM} as a function of different tuning parameters, the spin-orbit coupling ν in (a), the bulk superconducting gap Δ_0 in (b), and the surface magnetization strength M_z in (c).

with $N_y = 2N_x = 2N_z = 48$, which almost pushes to the limit of our numerical computing ability. An example is shown in Fig. 3 (d), where the blue dots represent numerical results, and could be fitted by an exponential function,

$$\psi_{MZM} \approx ae^{-r/\xi_{MZM}}. \tag{25}$$

with $r = x, y, z$. For the result in Fig. 3 (d), the black line is for the fitting results and shows

$$\xi_{MZM} = \begin{cases} \text{along x direction: } 2.0406, \\ \text{along y direction: } 1.92046, \\ \text{along z direction: } 1.10742, \end{cases} \tag{26}$$

in unit of lattice constants along different directions. We can see that the quantitative performance of the fitting is not as good as that of approach I. This comparison is made by examining Fig. 3 (b) [approach I] and Fig. 3 (d) [approach II].

We can further calculate the dependence of ξ_{MZM} on tuning parameters, including spin-orbit coupling v, superconducting gap Δ_0, and surface magnetism M_z. The results are shown in Fig. 5, which roughly agree with our prior analytical results in Eq. (23) despite the anisotropic behavior of ξ_{MZM}. As mentioned above, two cases are calculated for a comprehensive comparison, (1) considering only top surface magnetism [see Fig. 5 (a-c)]; (2) considering both top and bottom surface magnetism [see Fig. 5 (d-f)]. Since $N_z = 24$ is used in the simulation, it is much larger than the localization length of Majorana along z direction (~ 2). It leads to almost the same results for these two cases. Therefore, we only discuss the results in Fig. 5 (a-c). Similar to Eq. (26), we calculate the Majorana wave function along x, y, and z directions and obtain the fitting ξ_{MZM} along these three directions.

- Regarding the in-plane localization length (depicted as blue and red dots), Fig. 5 (a) suggests that they are nearly unaffected by variations in v. The observed inconsistency compared to the analytical results [see Eq. (23) and Fig. 2 (a)] may be attributed to the limitations of the fitting procedure.

- For the in-plane localization length (blue and red dots), we find that increasing Δ_0 [Fig. 5 (b)] or decreasing M_z [Fig. 5 (c)] give rise to the increase of ξ_{MZM}, consistent with both analytical results [see Eq. (23) and Fig. 2 (b-c)] and numerical results of approach I.

- For the z-direction localization length (orange dots), we find that increasing v [Fig. 5 (a)] or decreasing Δ_0 [Fig. 5 (b)] give rise to the increasing of ξ_{MZM}. However, it is almost unaffected by changing M_z as shown in Fig. 5 (c), since M_z is concentrated only on the sample's surface.

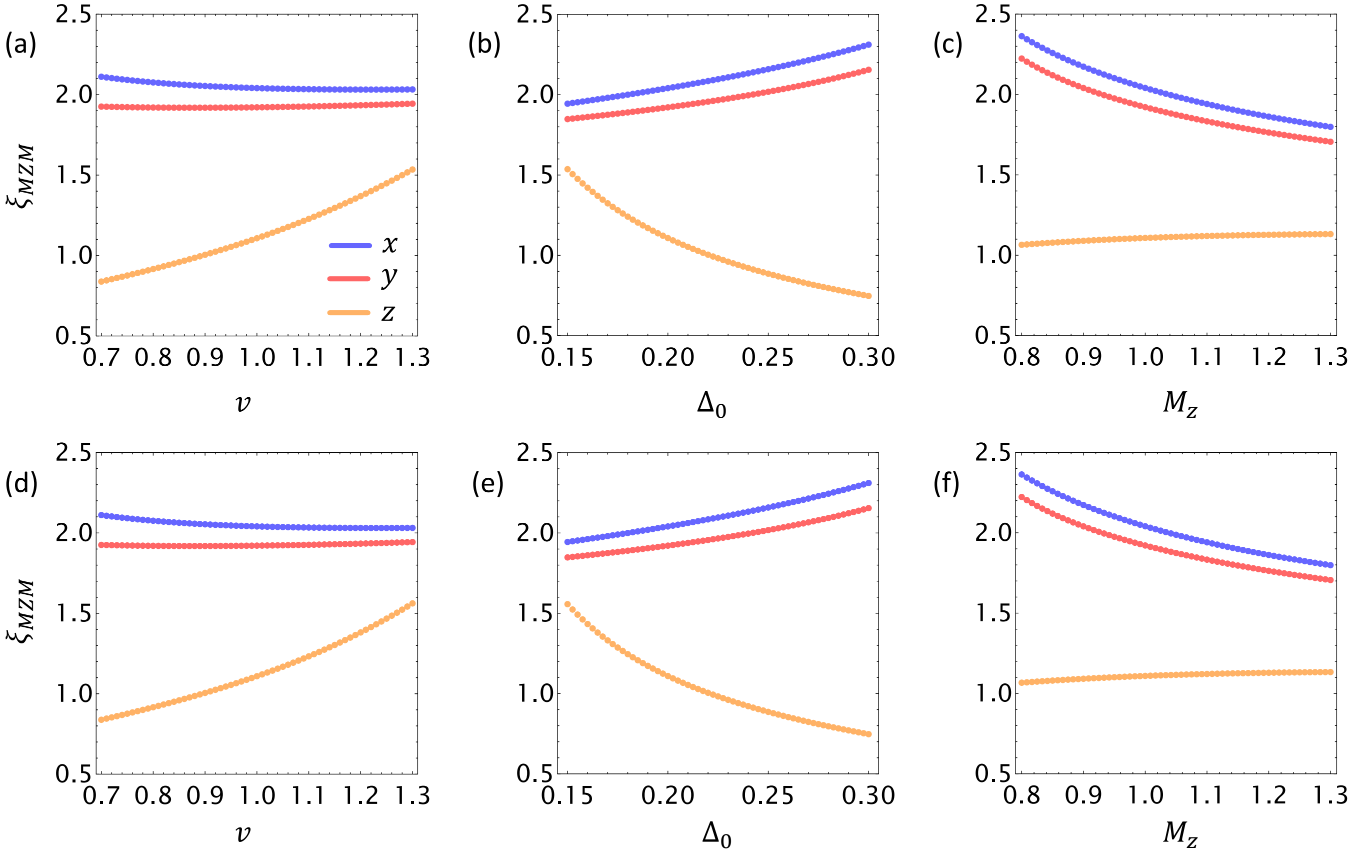

Supplementary Figure 5. **The numerical results for the localization length (ξ_{MZM}) of dislocation Majorana zero modes (MZMs) based on approach II.** Two scenarios are examined in numerical simulations: (1) only top surface magnetization (results in (a-c)); (2) both top and bottom surface magnetization (results in (d-f)). Similar to the numerical results of ξ_{MZM} shown in Fig. 6 above, we compute ξ_{MZM} in three directions, correlating with different tuning parameters: the spin-orbit coupling ν in (a), the bulk superconducting gap Δ_0 in (b), and the surface magnetization strength M_z in (c). The outcomes in (d), (e), (f) are entirely congruent with (a-c), respectively.

Supplementary Note. 2 Surface Topological Phase Diagram for $FeTe_{1-x}Se_x$: a First-Principles-Based Model Study

The main results discussed in the main text are based on an effective four-band model of a topological insulator. In this section, we reproduce some key results by using a eight-band $\mathbf{k}\cdot\mathbf{p}$ model fitted from a density-functional theory (DFT) calculated bands. The model, originally formulated by Ref. [3] is employed here for a comprehensive understanding. Below, we provide a detailed review of the model Hamiltonian to ensure this supplementary note is self-contained. First, Ref. [3] defined the four spinless basis as

$$|1\rangle = \frac{1}{\sqrt{2}}\left(\phi^A_{x^2-y^2} + \phi^B_{x^2-y^2}\right), \tag{27a}$$

$$|2\rangle = \frac{1}{2}\left[\left(\phi^A_{yz} + \phi^B_{yz}\right) + i\left(\phi^A_{xz} + \phi^B_{xz}\right)\right], \tag{27b}$$

$$|3\rangle = \frac{1}{2}\left[\left(\phi^A_{yz} + \phi^B_{yz}\right) - i\left(\phi^A_{xz} + \phi^B_{xz}\right)\right], \tag{27c}$$

$$|4\rangle = \frac{1}{\sqrt{2}}\left(\phi^A_{x^2-y^2} - \phi^B_{x^2-y^2}\right). \tag{27d}$$

where ϕ^A and ϕ^B are the 3d-orbitals of the Fe atom at site $A\left(-\frac{a}{4},\frac{a}{4},0\right)$ and site $B\left(\frac{a}{4},-\frac{a}{4},0\right)$, respectively. Inversion center is defined to interchange A and B sites, thus, $|1,2,3\rangle$ have an even parity and the basis $|4\rangle$ has an odd parity. Around Γ and Z, the effective model has full point group symmetry D_{4h} of the crystal. It contains three independent

generators: (1) inversion symmetry, (2) mirror symmetry, and (3) four-fold rotation around z axis R_{4z}. Based on the above basis, the representation matrices are given by

$$I = \begin{pmatrix} 1 & 0 & 0 & 0 \\ 0 & 1 & 0 & 0 \\ 0 & 0 & 1 & 0 \\ 0 & 0 & 0 & -1 \end{pmatrix}, \quad M_x = \begin{pmatrix} 1 & 0 & 0 & 0 \\ 0 & 0 & 1 & 0 \\ 0 & 1 & 0 & 0 \\ 0 & 0 & 0 & 1 \end{pmatrix}, \quad R_{4z} = \begin{pmatrix} -1 & 0 & 0 & 0 \\ 0 & -i & 0 & 0 \\ 0 & 0 & i & 0 \\ 0 & 0 & 0 & 1 \end{pmatrix}. \tag{28}$$

The four-band effective model $\mathcal{H}_0(\mathbf{k})$ without spin-orbit coupling (SOC) should satisfy $g\mathcal{H}_0(\mathbf{k})g^{-1} = \mathcal{H}_0(g\mathbf{k})$ with symmetry operator $g \in I, M_x, R_{4z}$. Up to k^2 order, the Hamiltonian is given by

$$\mathcal{H}_0(\mathbf{k}) = \begin{pmatrix} M_1(\mathbf{k}) & \gamma\sin(ck_z)(k_x - ik_y) & \gamma\sin(ck_z)(k_x + ik_y) & 0 \\ \gamma\sin(ck_z)(k_x + ik_y) & M_2(\mathbf{k}) & \beta(k_y^2 - k_x^2) + i\alpha k_x k_y & i\delta(k_x - ik_y) \\ \gamma\sin(ck_z)(k_x - ik_y) & \beta(k_y^2 - k_x^2) - i\alpha k_x k_y & M_2(\mathbf{k}) & i\delta(k_x + ik_y) \\ 0 & -i\delta(k_x + ik_y) & -i\delta(k_x - ik_y) & M_4(\mathbf{k}), \end{pmatrix} \tag{29}$$

where the diagonal terms are defined as

$$M_n(\mathbf{k}) = E_n + \frac{k_x^2 + k_y^2}{2m_{nx}} + t_{nz}[1 - \cos(ck_z)], \text{ for } n = 1, 2, 4. \tag{30}$$

Here, E_n is the energy of the band n at the Z point, while m_{nx} and t_{nz} are the in-plane effective mass and the z direction hopping of the band n, respectively. c is the lattice constant along the z direction. And $\alpha, \beta, \gamma, \delta$ are all inter-band hopping constants. Please note that the band inversion happens at the Z point, so that we will only focus on the model expanded around the Z point.

A. Eight-band model Hamiltonian and parameters

The full Hamiltonian with SOC takes the form

$$\mathcal{H}_{kp}(\mathbf{k}) = \mathcal{H}_0(\mathbf{k}) \otimes I_{2\times 2} + \mathcal{H}_{soc}(\mathbf{k}), \tag{31}$$

under the spinful bases $\{|1\rangle, |2\rangle, |3\rangle, |4\rangle\} \otimes \{|\uparrow\rangle, |\downarrow\rangle\}$. $I_{2\times 2}$ is a two-by-two identity matrix. The spinless part $\mathcal{H}_0(\mathbf{k})$ is given by Eq. (29). The SOC Hamiltonian $\mathcal{H}_{soc}$ contains two parts, on-site SOC and linear k_z terms. The on-site SOC between 3d orbitals and spin is given by

$$\mathcal{H}_{LS} = 2\lambda_1 L_z S_z + \lambda_2 \left(L^+ S^- + L^- S^+\right) \tag{32}$$

where $\mathbf{L}$ is the orbital angular momentum and $L_\pm = L_x \pm iL_y$, and $\mathbf{S}$ is the spin angular momentum and $S_\pm = S_x \pm iS_y$. In general, we have $\lambda_1 \neq \lambda_2$ due to the crystal anisotropy. We then transform the d-orbitals basis in Eq. (27) into the $|l, m\rangle$ representation (i.e., spherical harmonics), and

$$L_z|l, m\rangle = m|l, m\rangle, \tag{33a}$$

$$L_\pm|l, m\rangle = \sqrt{l(l+1) - m(m\pm 1)}|l, m\pm 1\rangle. \tag{33b}$$

This gives rises to

$$|1\rangle = \frac{1}{\sqrt{2}}\left(\phi^A_{x^2-y^2} + \phi^B_{x^2-y^2}\right) = \frac{1}{2}\left[(|2,2\rangle_A + |2,-2\rangle_A) + (|2,2\rangle_B + |2,-2\rangle_B)\right], \tag{34a}$$

$$|2\rangle = \frac{1}{2}\left[\left(\phi^A_{yz} + \phi^B_{yz}\right) + i\left(\phi^A_{xz} + \phi^B_{xz}\right)\right] = \frac{i}{\sqrt{2}}\left[|2,-1\rangle_A + |2,-1\rangle_B\right], \tag{34b}$$

$$|3\rangle = \frac{1}{2}\left[\left(\phi^A_{yz} + \phi^B_{yz}\right) - i\left(\phi^A_{xz} + \phi^B_{xz}\right)\right] = -\frac{i}{\sqrt{2}}\left[|2,1\rangle_A + |2,1\rangle_B\right], \tag{34c}$$

$$|4\rangle = \frac{1}{\sqrt{2}}\left(\phi^A_{x^2-y^2} - \phi^B_{x^2-y^2}\right) = \frac{1}{2}\left[(|2,2\rangle_A + |2,-2\rangle_A) - (|2,2\rangle_B + |2,-2\rangle_B)\right]. \tag{34d}$$

1.) For the on-site out-of-plane SOC $L_z S_z$. Those nonzero terms are,

$$\langle 2,\uparrow|2\lambda_1 L_z S_z|2,\uparrow\rangle = -\langle 2,\downarrow|2\lambda_1 L_z S_z|2,\downarrow\rangle = -\lambda_1, \tag{35a}$$

$$\langle 3,\uparrow|2\lambda_1 L_z S_z|3,\uparrow\rangle = -\langle 3,\downarrow|2\lambda_1 L_z S_z|3,\downarrow\rangle = \lambda_1. \tag{35b}$$

2.) For the on-site in-plane SOC $L^+S^- + L^-S^+$. Those nonzero terms are,

$$\langle 1,\uparrow|\lambda_2 L^- S^+|2,\downarrow\rangle = \langle 3,\uparrow|\lambda_2 L^- S^+|1,\downarrow\rangle = i\sqrt{2}\lambda_2. \quad (36)$$

In addition, the symmetry allowed linear k_z SOC terms are included to open the topologically nontrivial band gap in the $\Gamma - Z$ direction. Then, we have the SOC Hamiltonian,

$$\mathcal{H}_{soc} = \left(\begin{array}{cccc|cccc}
0 & 0 & 0 & 0 & 0 & i\sqrt{2}\lambda_2 & 0 & 0 \\
0 & -\lambda_1 & 0 & 0 & 0 & 0 & 0 & \sqrt{2}\lambda_3\sin(ck_z) \\
0 & 0 & \lambda_1 & 0 & i\sqrt{2}\lambda_2 & 0 & 0 & 0 \\
0 & 0 & 0 & 0 & 0 & 0 & \sqrt{2}\lambda_3\sin(ck_z) & 0 \\
\hline
0 & 0 & -i\sqrt{2}\lambda_2 & 0 & 0 & 0 & 0 & 0 \\
-i\sqrt{2}\lambda_2 & 0 & 0 & 0 & 0 & \lambda_1 & 0 & 0 \\
0 & 0 & 0 & \sqrt{2}\lambda_3\sin(ck_z) & 0 & 0 & -\lambda_1 & 0 \\
0 & \sqrt{2}\lambda_3\sin(ck_z) & 0 & 0 & 0 & 0 & 0 & 0
\end{array}\right). \quad (37)$$

In Ref. [3], Xu *et al.* fitted the parameters for the 8-band effective model $\mathcal{H}_{kp}(\mathbf{k})$. They are

$$\begin{aligned}
&E_1 = 0.226\ eV, \quad E_2 = 0.120\ eV, \quad E_4 = 0.275\ eV, \\
&m_{1x} = -0.271\ eV^{-1}\cdot \mathring{A}^{-2}, \quad m_{2x} = -0.151\ eV^{-1}\cdot \mathring{A}^{-2}, \quad m_{4x} = 0.131\ eV^{-1}\cdot \mathring{A}^{-2}, \\
&t_{1z} = -0.004\ eV, \quad t_{2z} = 0.076\ eV, \quad t_{4z} = -0.426\ eV, \\
&\alpha = 3.048\ eV\cdot \mathring{A}^2, \quad \beta = 1.524\ eV\cdot \mathring{A}^2, \quad \gamma = 0.003\ eV\cdot \mathring{A}, \quad \delta = 2.448\ eV\cdot \mathring{A}, \\
&\lambda_1 = 0.050\ eV, \quad \lambda_2 = 0.025\ eV, \quad \lambda_3 = 0.008\ eV.
\end{aligned} \quad (38)$$

Note that a few typo in the supplementary material of Ref. [3] has been corrected here. Moreover, we shift the energy level by $\Delta_E = 0.101036\ eV$, so that the chemical potential $\mu = 0$ corresponds to the energy of the surface Dirac cone. Namely, $\mathcal{H}_{kp}(\mathbf{k}) - \Delta_E \to \mathcal{H}_{kp}(\mathbf{k})$ will be used in the following calculations. As we discussed in the main text, we consider the surface ferromagnetism motivated by recent experiments [4–6]. It is described by

$$\mathcal{H}_{FM} = \mathrm{diag}[M_z, M_z, M_z, M_z, -M_z, -M_z, -M_z, -M_z]. \quad (39)$$

Please note that we only introduce it on the top and bottom surfaces of the sample, with a thickness chosen as 10 layers. To observe the magnetic gap on the surface Dirac cone, we perform a z-direction slab calculation. The spectrum along the k_x axis with $M_z = 0$ is shown in Fig. 6 (a), where the surface Dirac cone appears in a narrow energy windows $[-6.394, 6.831]\ meV$. At the Fermi level, other bands contribute to the bulk Fermi surface (not shown here, see Ref. [3] for details). As a comparison, the spectrum along the k_x axis in Fig. 6 (b) shows the gapped Dirac cones by surface magnetism with $M_z = 5\ meV$. We also show the localized wave function distribution of surface state in Fig. 6 (c), showing that the localization length is about 30 layers ($\sim 18\ nm$). The localization length is much larger than the typical length scale in Bi_2Se_3 and Bi_2Te_3 ($\sim 3\ nm$) due to the small SOC strength. This makes the full 3D simulation based on the above realistic parameters very challenging.

B. Superconducting band structures

We follow Ref. [3] by Xu *et al.* to include the superconducting pairings, and the Bogoliubov-de-Gennes (BdG) Hamiltonian of the system is

$$\mathcal{H}_{BdG}(\mathbf{k}) = \begin{pmatrix} \mathcal{H}_{kp}(\mathbf{k}) - \mu & \hat{\Delta}_s \\ \hat{\Delta}_s & -\mathcal{H}_{kp}(\mathbf{k}) + \mu \end{pmatrix} \quad (40)$$

where $\mathcal{H}_{kp}(\mathbf{k})$ also contains the surface magnetism and the spin-singlet pairing potential $\hat{\Delta}_s = \mathrm{diag}[\Delta_1, \Delta_2, \Delta_2, \Delta_1] \otimes I_{2\times 2}$. And $\Delta_1 = 2.5\ meV$ and $\Delta_2 = 1.7\ meV$ describe the superconducting gap of the $d_{x^2-y^2}$ and d_{xz} (d_{yz}) orbital, respectively. The full Nambu basis here is

$$\{c_{1,\mathbf{k}\uparrow}, c_{2,\mathbf{k}\uparrow}, c_{3,\mathbf{k}\uparrow}, c_{4,\mathbf{k}\uparrow}, c_{1,\mathbf{k}\downarrow}, c_{2,\mathbf{k}\downarrow}, c_{3,\mathbf{k}\downarrow}, c_{4,\mathbf{k}\downarrow}, -c^\dagger_{1,-\mathbf{k}\downarrow}, -c^\dagger_{3,-\mathbf{k}\downarrow}, -c^\dagger_{2,-\mathbf{k}\downarrow}, -c^\dagger_{4,-\mathbf{k}\downarrow}, c^\dagger_{1,-\mathbf{k}\uparrow}, c^\dagger_{3,-\mathbf{k}\uparrow}, c^\dagger_{2,-\mathbf{k}\uparrow}, c^\dagger_{4,-\mathbf{k}\uparrow}\}. \quad (41)$$

As we mentioned in the main text, the BdG Hamiltonian here is an ordinary s-wave superconductor model, and the bulk is indeed not topological. That means the superconducting state itself does not carry any nontrivial weak

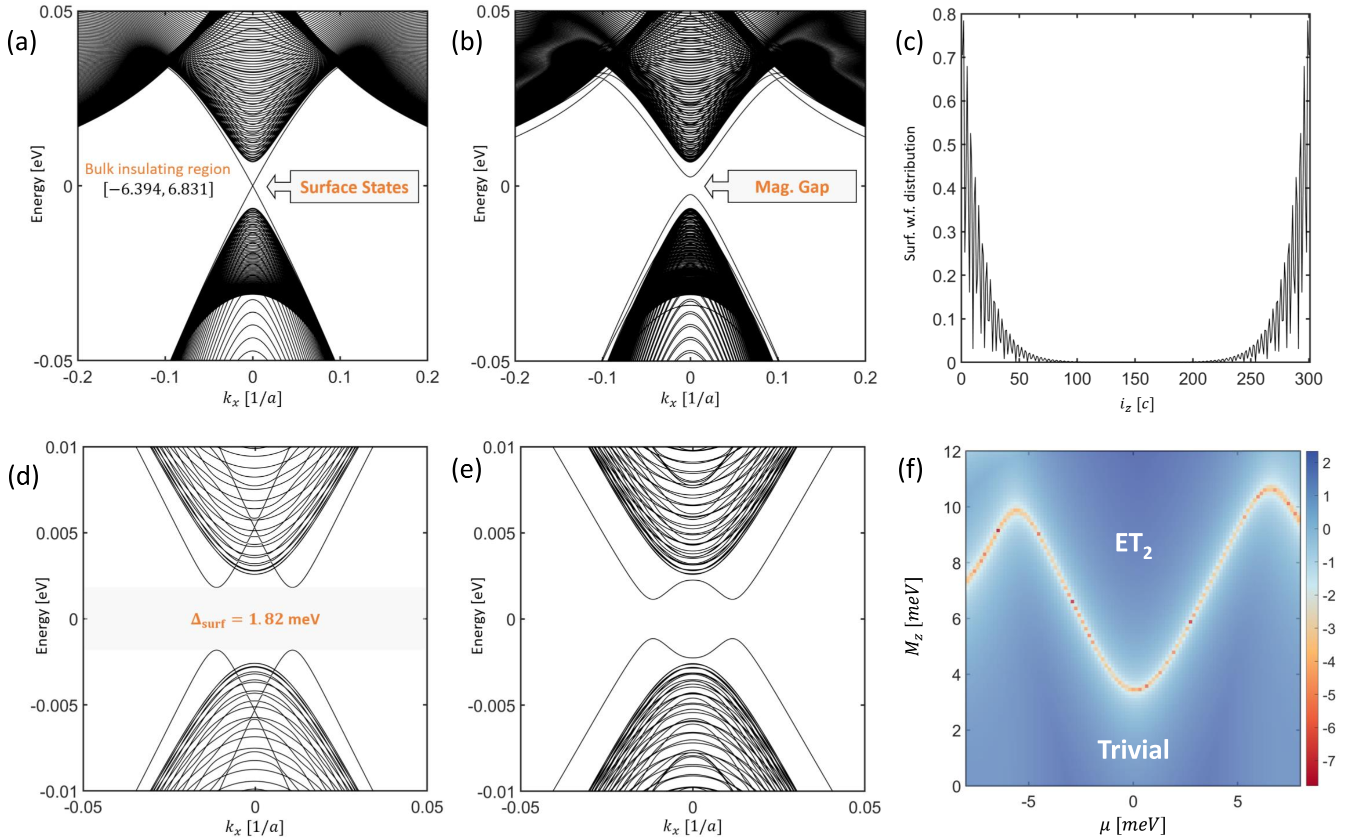

Supplementary Figure 6. **Band structures and surface topological phase diagram.** Based on the DFT-based model for $FeSe_{0.5}Te_{0.5}$, we first reproduce the topological surface states within a topological gap [−6.394, 6.831 meV], depicted in (a). Upon activating surface magnetization (M_z = 5 meV), a distinct magnetic gap emerges for the Dirac cone in (b). And (c) illustrates the localized wave function distribution of the Dirac surface states, the localization length in the c-axis is about 30 layers (∼ 18 nm). As FeSe0.5Te0.5 transitions into the superconducting phase, a proximity-induced superconducting gap (∼ 1.82 meV) emerges for the Dirac surface states, as illustrated in (d). The interplay between surface magnetism and superconductivity is evident in the spectrum depicted in (e). Additionally, the topological phase diagram, computed numerically with respect to chemical potential μ and M_z, is presented in (f). At low M_z, it exhibits a trivial phase and transforms into an embedded second-order topological superconductor housing 0D dislocation Majorana zero modes upon the increment of M_z. The color representation in (f) depicts the logarithm of the gap of the Dirac surface states at the Γ point. It is in excellent agreement with the results based on the effective model discussed in the main text (see Fig. 3 (b)).

$\mathbb{Z}_2$ index. The band structure without surface magnetism is shown in Fig. 6 (d), where the proximity induced superconducting gap is about 1.82 meV, consistent with recent experimental observations [7] that is slightly smaller than the bulk gaps. Furthermore, the time-reversal symmetry protected two fold degeneracy at the Γ point is broken after we turn on the surface magnetism that competes with the superconductivity, as shown in Fig. 6 (e) with $M_z = 5\ meV$. We will show it analytically later. These numerical results demonstrate the DFT fitted model is directly consistent to experiments, and the ET_2 phase proposed in this work has the potential to be realized in experiments.

C. Surface topological phase diagrams

We hope to first comment on the *infeasibility* of performing a full 3D simulation for the dislocation Majorana modes with the DFT-based model. Given the meV-level topological gap here, the Majorana localization length along the z direction is estimated to be around hundreds of unit cells. To avoid the finite-size effect here, a proper full 3D geometry could consist of millions of unit cells, leading to a BdG Hamiltonian matrix with a dimensionality of more than 10 million. Unfortunately, this is far beyond the capacity of our existing computing resources.

In spite of this challenge, the surface topological phase diagram manifests as an equivalent yet much more efficient

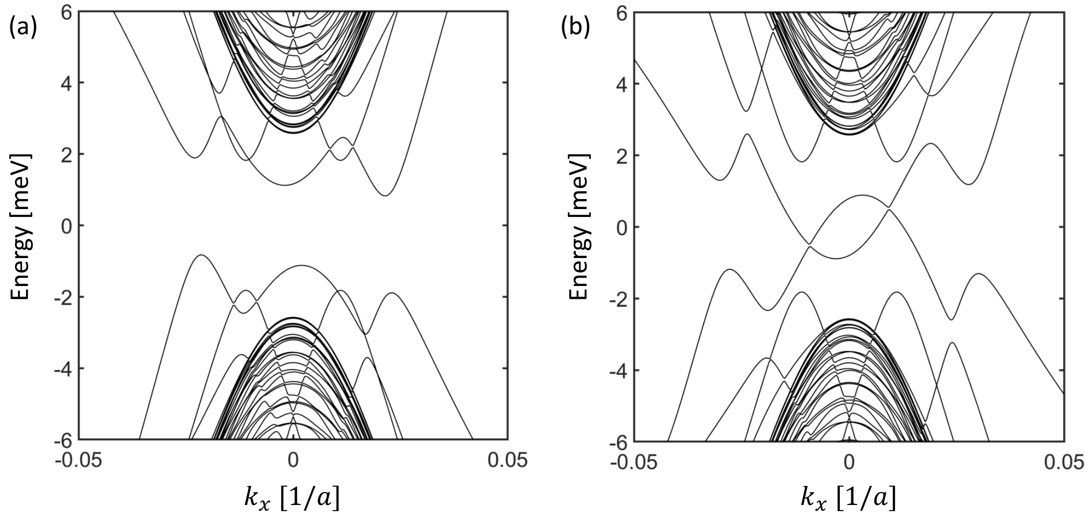

Supplementary Figure 7. Partial Fermi surface states based on DFT-fitted model. The interplay between in-plane magnetism and bulk superconductivity results in the closure of the gap in the surface spectrum. In (a), where $M_y = 26; meV$, the system remains gapped. Conversely, in (b), where $M_y = 32; meV$ is used, partial Fermi surface states become evident, illustrating the impact of stronger in-plane magnetism on the surface spectrum.

way to identify the existence of these dislocation Majorana modes, based on the topological theory we established in this work. The topological conditions have been clearly demonstrated via the low-energy effective theory. Then, we now use this DFT fitted $\mathbf{k}\cdot\mathbf{p}$ model to study the competition between bulk superconductivity and surface magnetism. We numerically map out the surface topological phase diagram as a function of chemical potential μ and magnetism strength M_z, and the results are shown in Fig. 6 (f). Despite its slight asymmetry of the phase boundary $\mu \to -\mu$ between trivial SC phase and topological embedded higher-order superconducting phase (ET_2), the phase diagram is almost reproducing the results based on the effective four-band model, presented in the main text. This asymmetry is caused by the topological gap for the surface Dirac cone, as mentioned above, which is in a narrow energy windows $[-6.394, 6.831]\ meV$.

As argued above, performing a full 3D simulation for the realistic DFT-fitted $\mathbf{k}\cdot\mathbf{p}$ model is computationally demanding. In the four-band model utilized in the main text, both spin-orbit coupling and superconducting gap are considerably larger than those in the realistic DFT-fitted $\mathbf{k}\cdot\mathbf{p}$ model. Consequently, the full 3D simulation in the main text is carried out for $N_x = N_y = 36$ and $N_z = 20$. However, for the DFT-fitted $\mathbf{k}\cdot\mathbf{p}$ model, we anticipate the necessity of $N_x = N_y = N_z \geq 200$, which surpasses our computational capabilities. Despite these limitations, the determination of topological phases relies on the features of gap closing and reopening. Therefore, we confidently conclude that the surface topological phase diagram presented in Fig. 6 (f) indeed confirms the ET_2 phase.

D. In-plane magnetism induced partial Fermi surfaces

While most experimental evidence suggests that the surface magnetism of $\mathrm{FeTe}_{1-x}\mathrm{Se}_x$ is predominantly oriented in the out-of-plane direction, we adhere to the approach in the main text and explore the interplay between in-plane magnetism and bulk superconductivity. This allows for a comparison between the results obtained from the effective four-band model and those derived from the DFT-fitted eight-band model. As detailed in the main text, when in-plane magnetism surpasses the superconducting gap of the surface states, it leads to the emergence of partial Fermi

surfaces. To illustrate this phenomenon, we consider the in-plane magnetism ($M_{\|} = (M_x, M_y)$) Hamiltonian as:

$$\mathcal{H}_{FM,\|} = \left(\begin{array}{cccc|cccc} 0 & 0 & 0 & 0 & M_x - iM_y & 0 & 0 & 0 \\ 0 & 0 & 0 & 0 & 0 & M_x - iM_y & 0 & 0 \\ 0 & 0 & 0 & 0 & 0 & 0 & M_x - iM_y & 0 \\ 0 & 0 & 0 & 0 & 0 & 0 & 0 & M_x - iM_y \\ \hline M_x + iM_y & 0 & 0 & 0 & 0 & 0 & 0 & 0 \\ 0 & M_x + iM_y & 0 & 0 & 0 & 0 & 0 & 0 \\ 0 & 0 & M_x + iM_y & 0 & 0 & 0 & 0 & 0 \\ 0 & 0 & 0 & M_x + iM_y & 0 & 0 & 0 & 0 \end{array}\right). \tag{42}$$

Without loss of generality, we consider M_x for illustration. The superconducting bands with $M_y = 26$ meV and 32 meV are shown in Fig. 7 (a) and Fig. Fig. 7 (b), respectively. The later one clearly shows the gapless bands that gives rise to the partial Fermi surface as discussed in the main text. We notice that a relative large in-plane magnetization is required (~ 30 meV), which may be not possible in current materials. It explains why fully gapped "U"-shaped local density of states is observed for $FeTe_{0.5}Se_{0.5}$ in experiments, otherwise a "V"-shaped local density of states is expected. Once again, we emphasize that all the results in this section aim to qualitatively and quantitatively "reproduce" the findings from the effective model presented in the main text. Furthermore, it is noteworthy that a recent experimen provides intriguing evidence of in-plane magnetization in a Josephson junction of $FeTe_{0.5}Se_{0.5}$ [8].

Supplementary Note. 3 Robustness of dMBS Against Magnetic Disorders

In this section, we consider the effect of magnetism fluctuations on the topological ET_2 phase. For the surface magnetism Hamiltonian defined in Eq. (39), we consider a spatial fluctuation $\widetilde{M}(\mathbf{r}) \in [-\Delta_M, \Delta_M]$ so that $M_z + \widetilde{M}(\mathbf{r})$ is used in Hamiltonian. For a full 3D lattice calculation, we set $\widetilde{M}$ as a random number on each lattice site to mimic the possible magnetic disorder effect that exists in FTS and other candidate systems. For one configuration, we obtain a few lowest energies: $\{E_{MZM}, E_1, E_2, E_3\}$. Then, an average of fluctuation configurations can be preformed, and yielding

$$E_{MZM}^{avg} = \frac{1}{N_{avg}} \sum_{\text{configurations}} E_{MZM}, \tag{43a}$$

$$E_1^{avg} = \frac{1}{N_{avg}} \sum_{\text{configurations}} E_1, \tag{43b}$$

$$E_2^{avg} = \frac{1}{N_{avg}} \sum_{\text{configurations}} E_2, \tag{43c}$$

$$E_3^{avg} = \frac{1}{N_{avg}} \sum_{\text{configurations}} E_3. \tag{43d}$$

Here N_{avg} is the number of fluctuation configurations. In the simulation, we use $N_y = 2N_x = 2N_z = 32$ and consider only top surface magnetism for simplicity. We also use $N_{avg} = 20$ which is large enough to test the robustness of dMBSs. The numerical results for dMBS (E_{MZM}^{avg}) and lowest bulk energies (E_2^{avg}, E_3^{avg} and E_4^{avg}) are shown in Fig. 8.

Remarkably, when $\Delta_{M_z} < 3M_z$ with $M_z = 1$, the system is highly robust against the magnetism fluctuations, as shown in Fig. 8. In fact, **the dislocation Majorana modes are spoiled only when Δ_{M_z} exceeds 5 times the value of M_z, which we believe is unlikely to happen in experiments.** This is because when M_z is extremely large, the instability of dMBSs is mostly due to the pair-breaking effect for the proximity-induced surface superconductivity. This limit, however, is inconsistent with the existing experimental observations for FTS by angle-resolved photoemission spectroscopy (see discussion of FTS in Sec. V in the main text), where surface magnetization indeed coexists with superconductivity. Therefore, this numerical result clearly proves the robustness of dMBSs against the magnetism fluctuations in real space. This is why we are confident that our proposal will be realized in experiments very soon.

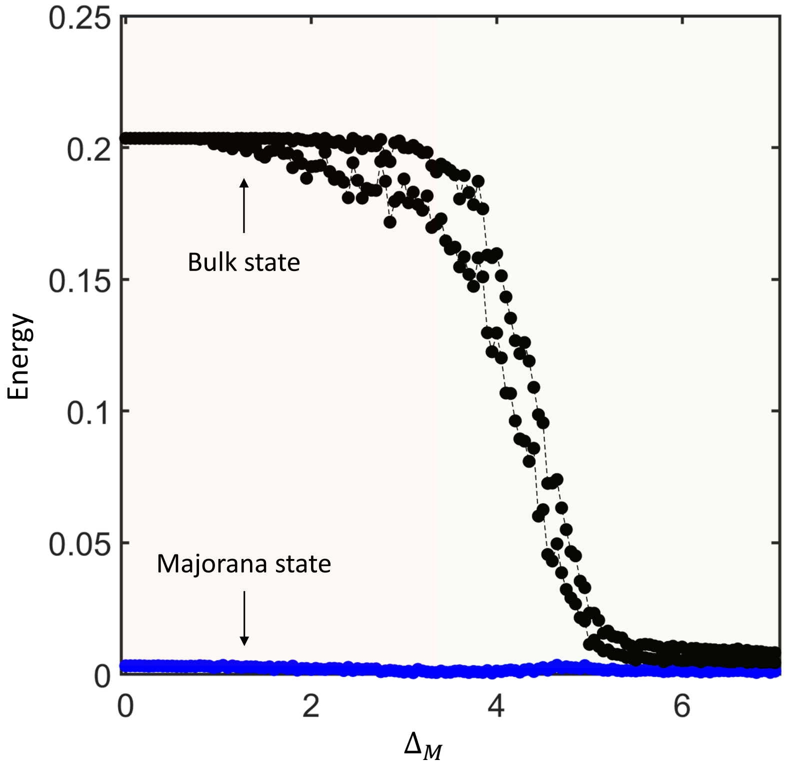

Supplementary Figure 8. **Robustness of dislocation Majorana bound states.** We consider a spatial magnetization fluctuation $\widetilde{M} \in [-\Delta_{M_z}, \Delta_{M_z}]$ in the full 3D lattice calculation for the energy of dMBS (blue dots) and bulk states (black dots). Specifically, we focus on the z-component magnetization and find dMBSs are stable before Δ_{M_z} exceeds 5 times the value of M_z. This critical value means the breaking down of superconductivity.

Supplementary References

[1] C.-X. Liu, X.-L. Qi, H. Zhang, X. Dai, Z. Fang, and S.-C. Zhang, Model hamiltonian for topological insulators, Phys. Rev. B **82**, 045122 (2010).

[2] Y. Ran, Y. Zhang, and A. Vishwanath, One-dimensional topologically protected modes in topological insulators with lattice dislocations, Nature Physics **5**, 298 (2009).

[3] G. Xu, B. Lian, P. Tang, X.-L. Qi, and S.-C. Zhang, Topological superconductivity on the surface of fe-based superconductors, Phys. Rev. Lett. **117**, 047001 (2016).

[4] N. Zaki, G. Gu, A. Tsvelik, C. Wu, and P. D. Johnson, Time-reversal symmetry breaking in the fe-chalcogenide superconductors, Proceedings of the National Academy of Sciences **118** (2021).

[5] N. J. McLaughlin, H. Wang, M. Huang, E. Lee-Wong, L. Hu, H. Lu, G. Q. Yan, G. Gu, C. Wu, Y.-Z. You, and C. R. Du, Strong correlation between superconductivity and ferromagnetism in an fe-chalcogenide superconductor, Nano Letters **21**, 7277 (2021).

[6] C. Farhang, N. Zaki, J. Wang, G. Gu, P. D. Johnson, and J. Xia, Revealing the origin of time-reversal symmetry breaking in fe-chalcogenide superconductor fete$_{1-x}$se$_x$, Phys. Rev. Lett. **130**, 046702 (2023).

[7] P. Zhang, K. Yaji, T. Hashimoto, Y. Ota, T. Kondo, K. Okazaki, Z. Wang, J. Wen, G. Gu, H. Ding, *et al.*, Observation of topological superconductivity on the surface of an iron-based superconductor, Science **360**, 182 (2018).

[8] G. Qiu, H.-Y. Yang, L. Hu, H. Zhang, C.-Y. Chen, Y. Lyu, C. Eckberg, P. Deng, S. Krylyuk, A. V. Davydov, *et al.*, Emergent ferromagnetism with superconductivity in fe (te, se) van der waals josephson junctions, Nature Communications **14**, 6691 (2023).
